# Smoking and body weight: evidence from China health and nutrition survey

**DOI:** 10.1186/s12889-015-2549-9

**Published:** 2015-12-14

**Authors:** Qing Wang

**Affiliations:** School of Business, Dalian University of Technology, No.2 Dagong Road, Liaodong Bay New District, Panjin, 124221 Liaoning China

**Keywords:** Smoking, Body weight, Number of cigarettes consumed daily, China

## Abstract

**Background:**

The effects of cigarette smoking on body weight remain inconclusive. This study evaluated this relationship using the latest data from China, the largest consumer market of tobacco in the world, which is also experiencing a steady increase in patients with obesity.

**Methods:**

Using data obtained from China Health and Nutrition Survey (CHNS) 1991–2011, Logit Model and Two-Stage Residual Inclusion (2SRI) estimation were applied. Local tobacco yield was considered as instrument variable for smoking behavior and corrected for endogeneity.

**Results:**

Smoking increased the likelihood of being underweight by 0.9 % and healthy weight by 5.3 %, while the likelihood of overweight and obesity decreased by 6.5 %, of which obesity reduced by 5.1 %. After correcting for endogeneity, the results were consistent and stronger. Cigarette smoking increased the likelihood of being underweight by 2.7 % and healthy weight by 12.7 %, while it decreased the likelihood of overweight and obesity by 13 %, of which obesity reduced by 10 %.

**Conclusion:**

Smoking induced heterogeneous effects on body weight. Smoking had a positive effect on underweight and healthy weight, while a negative effect on overweight and obesity. Tobacco control interventions may lead to weight loss among healthy subjects, while the effects on obese subjects were not obvious as expected.

## Background

China was previously considered to be one of the countries with the leanest population. However, China has quickly caught up with the West [1]. Estimates from China Health and Nutrition Survey (CHNS) 1991–2011 indicated that the prevalence of overweight adults nearly doubled.

Behavioral risks are a major contributor to obesity. There is a considerable interest in understanding the effects of smoking on body weight. The exact nature remains unclear due to the mixed results observed in the literature review. The direction and magnitude of these effects varied across studies. Some studies reported that smoking was associated with lower weights and body mass index (BMI), whereas some studies indicated a conflicting effect of significantly increased BMI [2–11]. A few disturbing results indicate no evidence for the claim that smoking behavior contributed to the change in obesity [12, 13]. The recent studies reconciled an inverse average effect of smoking on body weight. Effects of smoking on body weight may be differences across BMI groups [14–16].

Biologically, there is a general perception that smoking decreases body weight due to reasons like decrease in appetite and calorie intake, enhanced metabolism, and reduced fat accumulation. This may be due to the effects of nicotine on brain’s regulation of appetite and energy expenditure [17, 18]. However, smoking decreases exercise by constraining respiratory functions [19]. Smoking counteracts the previously mentioned effects on appetite and metabolism and results in increased body weight. Therefore, the biologic pathways suggest an ambiguous net effect of smoking on body weight.

Moreover, these conflicting results may be because of data differences and model specifications [16]. More empirical evidence is needed to figure out the association between smoking and body weight [2]. The previous studies on the relationship have mainly focused on the US population. Only one study by Fang et al. using cross-sectional data in 2006 has analyzed this issue in the Chinese population [15]. The results in Fang et al. *study* did not provide consistent estimates [15]. This study aims to explore these issues with latest panel data from China controlling unobserved variables and endogeneity.

## Methods

### Data

Data was obtained from CHNS, 1991–2011, which is publicly available (http://www.cpc.unc.edu/projects/china/data/datasets/longitudinal). The researchers from the Carolina Population Center who are responsible for the collection of CHNS data had received ethic approval the University of North Carolina at Chapel Hill. CHNS was a longitudinal survey mainly covering Guangxi Zhuang and eight other provinces with substantial variations in terms of geography, economic development, public resources, and health indicators in the years 1991, 1993, 1997, 2000, 2004, 2006, 2009, and 2011. The nine provinces accounted for approximately 45 % of China’s population in 2011. The survey collected data on detailed information on measures of health behaviors and corresponding outcomes, such as smoking, drinking, height, and weight.

A multistage, random cluster process was included to draw the sample surveyed in each province. Counties in the nine provinces were stratified by income level (low, middle, and high). Four counties were randomly selected from each province by a weighted-sampling scheme (1 in low, 2 in middle, and 1 in high-income levels). In addition, the provincial capital and the lowest-income city were also included. Villages and townships within the counties and urban and suburban neighborhoods within the cities were selected randomly. There were about 4400 households in the survey, covering about 19,000 individuals in the most recent wave. A detailed description of the design of the CHNS is accessible at http://www.cpc.unc.edu/projects/china.

### Variables

#### Weight status variables

Height and weight for each respondent, included in the CNHS dataset, were measured by a health professional during the interview. Detailed information including the instruments used to measure height and weight, calibration status of the instrument and other related information could be accessed by http://www.cpc.unc.edu/projects/china/data/questionnaires/Training_Manual_2006_short.pdf. These physical parameters provided us with data of the actual height and weight of the individuals. Burkhauser and Cawley noted that there was a significant bias between data on self-reported and actual weight, particularly in overweight and obese subjects who tend to under-report body weight [20]. The BMI was calculated for each respondent with actual height and weight, avoiding measurement errors or reporting bias. Since there may be a non-linear relationships between smoking and body weight, four category variables were constructed according to the Asian cut offs providing by WHO: underweight (BMI < 18.5), healthy weight (18.5 < =BMI < 22.9), overweight (23 = <BMI), of which obese(25 = <BMI) [21].

### Cigarette smoking variable

In the CHNS questionnaire, respondents were asked if they were active smokers at the time of the survey. The respondents who answered “yes” were further asked on the number of cigarettes smoked per day. Two variables were constructed to measure smoking behavior. The first was smoking status, which was classified into smoker or non-smoker. The second was log of “number of cigarettes consumed daily.” Such a continuous measure of smoking could capture the intensity of smoking dependence among respondents, which may not be obtained in the present widely used binary indicator of being a current smoker.

### Socio-economic status, health behaviors as well as demographic characters

Variables representing social and economic status were controlled. Education was defined at three levels: primary school or below, junior high school and senior high school or above. Two dummy variables for education were included, with “primary school or below” as the reference group. Job status was also a dummy variable, and “unemployed” was used as the reference group. Another dummy variable “living in urban area” indicated whether the individual lived in an urban area or not. Log of household per capita income, which was deflated by the consumer price index, was controlled.

Alcoholic behavior was included as an indicator of respondents’ current health behaviors. Frequency of alcohol intake was questioned and was considered to evaluate the alcoholic status. People who answered “almost every day and 3–4 times a week” was coded as a frequent drinker; “once or twice a week” was regarded as less-frequent drinker; and “once or twice a month” was included as barely drinking; “no more than once a month and never drink” was coded as non-drinker (reference group).

Demographic variables such as gender (reference group: female) and age were concluded. “Age” was a continuous variable and age quadric was included in the model to capture the non-linear impact of age on the dependent variable. State-and time-fixed effects were also included.

### Estimation strategy

Since body weight measures were category variables, Logit model was estimated [22]. The regression equation was

$$ W={\upbeta}_0+s{\upbeta}_1+x{\upbeta}_2+\upvarepsilon $$

where *w:* the body weight category variable;

*s:* smoking behavior;

*χ*: a vector of other explanatory variables;

ε: a residual term; and

β_0_ β_1_ β_2_: coefficients to be estimated

Body weight may have reverse effects on smoking behavior if individuals select to smoke in order to control their weight, or if they quit smoking due to health risk concern [23]. Two-stage least squares (2SLS) estimation could not be performed to solve the endogeneity problem due to categorical nature of the dependent variables. The two-stage residual inclusion (2SRI) estimation extends the 2SLS linear modeling framework with dependent variable in the second stage to be non-linear outcomes. 2SRI estimation has been widely applied to address endogeneity in health economics and health services research [24], which showed that 2SRI yields consistent and efficient results [25]. Following the research of Terza [24], 2SRI Model was estimated. The first-stage equation was estimated as follows:

1 $$ s={\upalpha}_0+IV{\upalpha}_1+x{\upalpha}_2+u $$

where

*IV*: instrumental variable;

*u:* residual;

α_0_α_1_α_2_: coefficients to be estimated

The instrumental variable for smoking behavior was log of province-level tobacco yield. Province-level tobacco yield’s variation ranged from 0.1 to 71.1 million tons; the mean value was 15.71 million tons. Tobacco production levels varied substantially across geographic areas during the period of 1991–2011. This instrumental variable should be correlated with smoking status [22]. Some tobacco control strategies targeted the tobacco companies and farmers to restrict the tobacco consumption [26]. Crop substitution for tobacco farmers as a way to reduce tobacco yield has met with success, as demonstrated in Brazil [27] and Bolivia [28]. As expected, tobacco yield in provinces has been positively correlated with the smoking behavior (Appendix). Also, the instrumental variable for smoking should not be directly affecting body weight. It only works through making effects on smoking status indirectly, which cannot be proved by data analysis directly. But the instrumental variable was jointly significant at the 1 % level in an F-test, indicating the validity of our instrument.

For smoking status was the dependent variable, Logit model was applied in the first stage; As number of cigarettes consumed daily used to measure smoking behavior, Ordinary least square (OLS) model was applied to estimate the effects of local tobacco yield on smoking behavior.

The second-stage equation can be obtained by adding the residual from the first stage into equation (1) as follows:

2 $$ w={\upbeta}_0+s{\upbeta}_1+x{\upbeta}_2+\widehat{u}{\upbeta}_3+\upvarepsilon $$

where *û* : the fitted residual from the first stage

Because the nonlinear system was estimated in two steps, the associated standard errors are incorrect, as they fail to account for the stochastic nature of the estimated residual terms. Therefore, we used bootstrapping with 1000 iterations to calculate correct standard errors [24]. Holding all other variables in the model at their means, marginal effects were reported in the results section to ascertain the magnitude of the effects of smoking behavior on body weight. Stata 12 was used for statistical calculations.

## Results

### Descriptive statistics

**Fig. 1:**
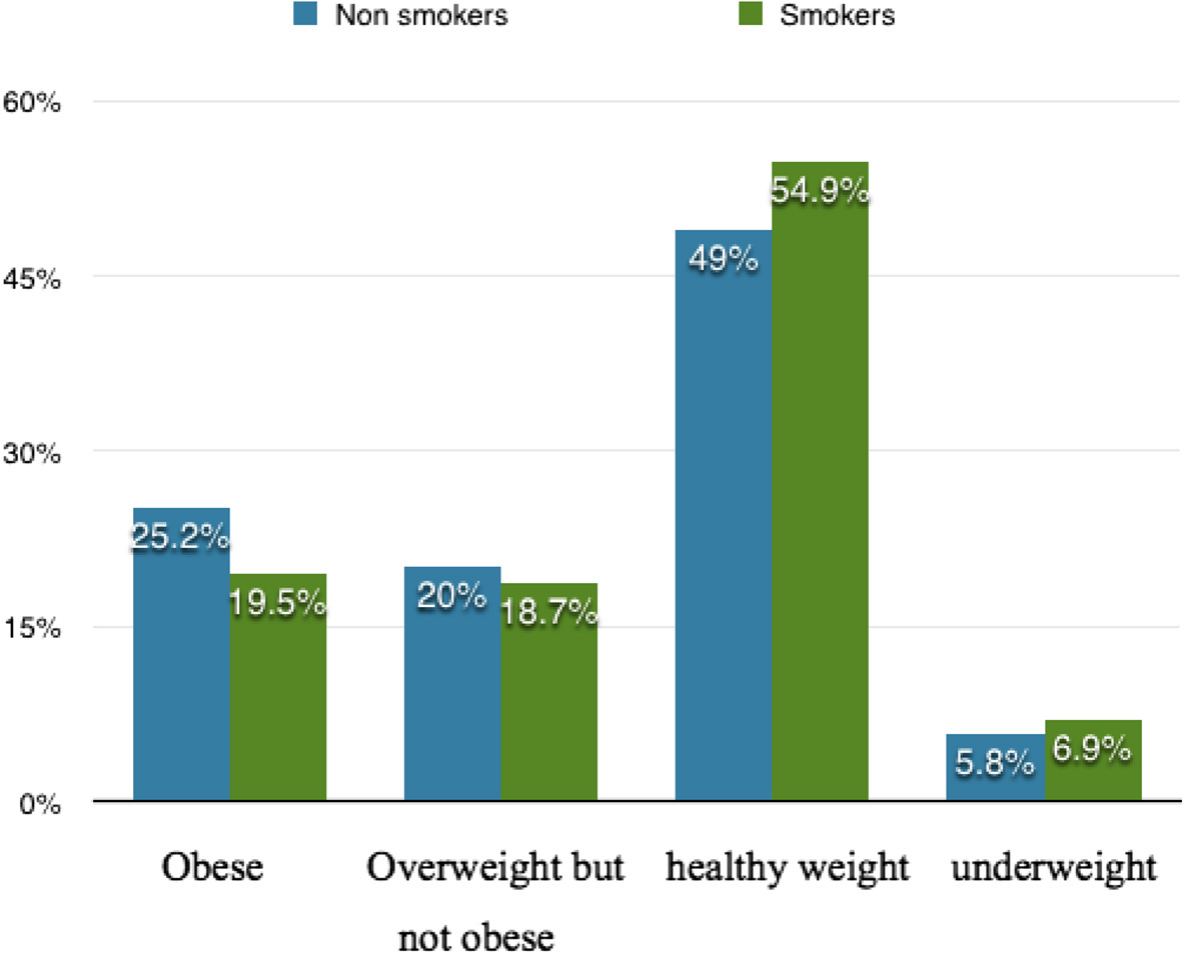
Percentage of body weight categories by smoking status

Table 1 shows descriptive statistics for the variables by smoking status. The final sample size was 70,394. The average BMI was 22.85, which indicated that our study subjects were of healthy weight in general. More specifically, 6.1 % were underweight (BMI < 18.5), 50.8 % were in the range of healthy weight (23 > BMI > =18.5), 19.6 % were overweight but not obese (23 = <BMI < 25), and 23.5 % were obese (BMI > =25). On average, adults smoked 4.7 cigarettes per day and the smoking rate was 30.4 %. *χ*2 test presented the differences of body weight across smoker and non-smoker were significance. Figure 1 shows that compare to non-smokers, the percent of current smokers be healthy weight and underweight were higher, while the percent of be obese and overweight for smokers were lower.

**Table 1:** Characteristics of the survey respondents by smoking status

Variables	Total	Smoker	Non-smoker
	*n* = 70,394	*n* = 21,340	*n* = 49,054
BMI^#^	22.85 (3.33)	22.47 (3.15)	23.02 (3.4)
Underweight	6.1 %	6.9 %	5.8 %
Health weight	50.8 %	54.9 %	49.1 %
Overweight but not obese	19.6 %	18.7 %	19.9 %
Obese	23.5 %	19.5 %	25.2 %
Current smoker	30.4 %	100 %	0
Number of cigarettes consumed daily^#^	4.7 (8.7)	15.59 (8.95)	0
Age^#^	46.6 (15.6)	46.7 (14.5)	46.5 (16.1)
Gender	48.1 %	92.6 %	28.6 %
Urban	31.8 %	28.2 %	33.4 %
Employed	72.8 %	63.3 %	76.9 %
Household income per capita^#^	7548 (10824)	7048 (10455)	7767 (10996)
Education level			
Primary school and below	46.5 %	42.9 %	48.1 %
Secondary school	30.9 %	34.9 %	29.1 %
High school and above	22.6 %	22.2 %	22.8 %
Drinking behavior			
Drink-frequently	10.2 %	24.3 %	4.3 %
Drink less frequently	13.2 %	27.6 %	6.9 %
Drink barely	6.6 %	11.3 %	4.5 %
Non-drinker	70 %	36.7 %	84.3 %

Note: #Mean, SD;

Table 2 shows the multivariate regression results for the effects of smoking status on body weight. Socio-economic status, health behaviors as well as demographic characters were controlled, but without addressing the endogeneity of smoking status. The smoking status was positively associated with being overweight and obese, negatively associated with being underweight and healthy weight.

**Table 2:** Effects of smoking behavior on different level body weight using the logit model

Dependent variable	Effects of current smoker			Effects of number of cigarettes consumed daily		
	Coefficient	S.E.	95 % C.I.	Coefficient	S.E.	95 % C.I.
Underweight	0.169***	0.045	(0.08, 0.258)	0.042**	0.016	(0.01, 0.073)
Healthy Weight	0.213***	0.022	(0.17, 0.257)	0.079***	0.008	(0.063, 0.095)
Over weight	−0.263***	0.022	(-0.305, -0.22)	−0.093***	0.008	(-0.109, -0.077)
Obese	−0.275***	0.023	(-0.321, 0.229)	-0.113***	0.01	(-0.133, -0.094)

Note:

** significant at the 5 % level;

*** significant at the 1 % level

Std. errors are robust

Underweight (BMI < 18.5), healthy weight (18.5 < =BMI < 22.9), overweight (23 = <BMI), obese(25 = <BMI)

Socio-economic status, health behaviors as well as demographic characters, year dummy and district dummy are included. The full results are available from the authors upon request

To perform the 2SRI estimation, the determinants of smoking behavior was estimated. Our instrument (province-level tobacco yield) and other variables described above were applied to address the endogeneity issue (Appendix). When estimated with 2SRI, the effects of smoking status on body weight were consistent and the residuals inserted into the second stage were highly significant (Table 3), indicating that smoking status was endogenous (Appendix).

**Table 3:** Effects of smoking behavior on different level body weight using the 2-stage Residual Inclusion estimation (2SRI)

	Effects of current smoker			Effects of number of cigarettes consumed daily		
	Coefficient	S.E.	95 % C.I.	Coefficient	S.E.	95 % C.I.
The dependent variable was underweight dummy variable						
Underweight	0.43***	0.134	(0.168, 0.692)	0.238***	0.044	(0.015 0.325)
Residual	-0.12*	0.069	(-0.255, 0.015)	-0.029***	0.06	(-0.041 -0.016)
The dependent variable was healthy weight dummy variable						
Healthy Weight	0.511***	0.062	(0.388, 0.634)	0.133***	0.021	(0.092 0.175)
Residual	-0.167***	0.031	(-0.228.-0.106)	-0.008** .	0.003	(-0.014 -0.002)
The dependent variable was overweight dummy variable						
Over weight and obese	-0.538***	0.063	(-0.661, -0.414)	-0.199**	0.022	(-0.242 -0.156)
Residual	0.141***	0.03	(0.082, 0.200)	0.015*	0.003	(0.001,0.021)
The dependent variable was obese dummy variable						
Obese	-0.596***	0.063	(-0.721, -0.471)	-0.227***	0.025	(-0.277 -0.177)
Residual	0.162***	0.0309	(0.102, 0.223)	0.017** .	0.003	(0.010 0.024)

Note:*significant at the 10 % level;

**significant at the 5 % level;

***significant at the 1 % level

Std. errors are robust

Underweight (BMI < 18.5), healthy weight (18.5 < =BMI < 22.9), overweight (23 = <BMI), obese(25 = <BMI)

Socio-economic status, health behaviors as well as demographic characters, year dummy and district dummy are included. The full results are available from the authors upon request

Table 4 shows the marginal effects of smoking behavior on body weight. The marginal effects without addressing the endogeneity issue were as follows:

**Table 4:** Marginal effects of smoking behavior on health outcomes using logit and 2SRI regression

	Marginal effects of current smoker		Marginal effects of number of cigarettes consumed daily	
	Logit Model	2SRI Model	Logit Model	2SRI Model
Underweight	0.009***	0.027***	0.002***	0.012***
Healthy weight	0.053***	0.127***	0.020***	0.033***
Overweight	-0.065***	-0.13***	-0.024***	-0.039**
Obese	-0.051***	-0.10***	-0.020***	-0.039***

Note:

**significant at the 5 % level;

***significant at the 1 % level

Std. errors are robust

Underweight (BMI < 18.5), healthy weight (18.5 < =BMI < 22.9), overweight (23 = <BMI), obese (25 = <BMI)

Socio-economic status, health behaviors as well as demographic characters, year dummy and district dummy are included

- *Cigarette smoking increased the likelihood of being underweight by 0.9 %, healthy weight by 5.3 %.*
- *Cigarette smoking decreased the likelihood of being overweight by 6.5 %, of which obese by 5.1 %.*
- *When respondents smoked a cigarette more per day, the likelihood of being underweight was increased by 0.2 %, healthy weight by 2 %.*
- *When respondents smoked a cigarette more per day, the likelihood of being overweight was decreased by 2.4 %, of which obese by 2 %.*

But when estimated with 2SRI, the marginal effects of smoking status were more than that on body weight. More specifically, we found that

- *Cigarette smoking increased the likelihood of being underweight by 2.7 % and healthy weight by 12.7 %.*
- *Cigarette smoking decreased the likelihood of being overweight by 13 %, of which obese by 10 %.*
- *When respondents smoked a cigarette more, the likelihood of being underweight was increased by 1.2 % and healthy weight by 3.3 %.*
- *When respondents smoked a cigarette more, the likelihood of being overweight and obese was decreased by 3.9 %, of which obese by 3.9 %.*

Basically, these marginal effects were significant at the 5 % or 1 % levels (see Table 4).

## Discussion

Smoking was positively association with the likelihood of being underweight and healthy weight, but negatively related to the likelihood of being overweight and obesity in China. Smoking induced heterogeneous effects on body weight, which supported that the relationship between smoking and body weight is not linear [6]. The linear model was applied to estimate smoking effects on BMI, which essentially represents the average effects of smoking across the BMI distribution. Depending on the sample characteristics, model specification and estimation, the observed differences in “mean effects” between these studies which applied linear model may be due to the masked effect of heterogeneity at BMI distribution.

Besides weight-related biological processes mentioned by Wehby et al., potential interactions between smoking and weight-related preference may jointly contribute to the heterogeneous results [3, 8, 16]. Some individuals may take up smoking as a method of weight control in order to be slim or some others may reduce smoking due to health risks caused by obesity [10, 11]. Thus, smoking may make negative effects at high BMI levels but not at low/moderate BMI levels.

Endogeniety arises due to reverse causality between body weight and smoking, which may result in regression bias. Considering the endogenous selection of smoking, the 2SRI estimation approach was applied. Province-level tobacco yield was involved as instrument variable, which was proved to be valid. The residuals inserted into the second stage were highly significant, indicating that smoking was endogenous. The effects of smoking on body weight were consistent with estimations after solving the endogeneity issue. In the single equation estimates that do not correct for endogeneity, the relationship between smoking behavior and body weight were weaker. It implied that overweight respondents may control smoking and weight to lead a healthy life, while underweight respondents may be more likely to take up smoking as a method of weight control, and may even ignore the health risk accompanied with smoking [16].

The current high prevalence of smoking among adults and the rise of body weight in China have become major public health concerns. Our results have several health policy implications for China. First, an integrated tobacco control policy should be based on the non-linear relationship between smoking behavior and body weight. When the aim is to reduce the overall smoking rates, an increase in obesity rates among the general population could be of less concern for policy instruments. Indeed, the general health status of population would be improved with decreased smoking rates, which may more than offset the potentially modest weight gain. Second, body weight disparity may be in part related to smoking status. Policies aimed at achieving body control should recognize the role of smoking behavior. Policy makers should discourage individual to regard smoking as body control management strategy and figure out effective and healthy measures to control body weight.

This study contributes to the body of literature in two aspects. First, this is the first study to quantify relationships between smoking behavior and body weight using panel data from China, in which the unobserved variables can be controlled. Smoking and body weight are closely associated with state tobacco control policy, individuals’ preferences toward health and risk taking, and these factors are typically unobserved variables [16]. The estimation of smoking effects may be considered biased for ignoring the roles of these variables [22]. Panel data involved in this study could help to control unobserved variables. Second, latest data was used in this study to estimate the effects of smoking on body weight. The dataset involved in only one study by Fang et al. analyzed the effects of smoking on body weight in China was almost ten years age (year 2006) [15]. In recent years, obesity dramatically rises; China government has signed on to the WHO framework convention on tobacco control in January 2006 and committed itself to control smoking [29, 30]. The latest data should be involved and studied.

The manuscript was not without limited. Firstly, “selection effect”, a result of selective processes across body weight group, might lead to regression bias. There may be substantial differences in smoking characteristics between obese/overweight cohort and underweight/healthy weight cohort. Selection effects refer to the possibility that individuals with specific body weight tend to quit smoking [31]. A limitation of CHNS public-use datasets is that they did not allow us to identify former smokers’ body weight status while he/she quitted smoking, so we were not able to control for former smokers’ body weight status in smoke quitting period that might lead to selection effect. Second, energy intake was not included in this study, which was believed to contribute to the heterogeneous effects of smoking on body weight [16]. Even though CHNS has information on caloric intake, we found irreconcilable discrepancies. For example, the underweight population reported high caloric consumption and the obese population reported low caloric consumption.

## Conclusion

Smoking resulted in an increase of being underweight and healthy weight, an decrease of being overweight and obese. Smokers tended to have a healthy weight than non-smokers. From a policy perspective, tobacco control activities may lead to weight gain among subjects of underweight and healthy weight, while the effects on obese subjects may be the opposite, and a large increase in obesity prevalence rates is not expected. Understanding the relationship between smoking and body weight across body weight group can help tailor interventions for specific group of smokers.

## Appendix

**Table 5:** The impact of smoking behavior on body weight: results from the first stage of 2SRI

	Number of cigarettes consumed daily on body weight (OLS model)		Current smoker (Logit model)	
	Mean	Std. Dev.	Mean	Std. Dev.
Local tobacco yield	0.053***	0.008	0.023***	0.003
Age	0.079***	0.004	-0.000	0.00
Age quadric	-0.001***	0.000	-0.001***	0.000
Gender	3.228***	0.033	1.144***	0.01
Urban	-0.154***	0.026	-0.059***	0.009
Employed	0.122***	0.029	0.058***	0.01
Log of household income per capita	-0.53***	0.093	-0.164***	0.031
Education level				
Secondary school	0.447***	0.032	0.178***	0.012
High school and above	0.284***	0.029	0.146***	0.011
Drink behavior				
Drink frequently	1.112***	0.034	0.737***	0.019
Drink less frequently	0.986***	0.030	0.521***	0.016
Drink barely	0.691***	0.038	0.277***	0.02

Note:

*** significant at the 1 % level

Std. errors are robust

Year dummy and district dummy were included

## Abbreviations

CHNS: China health and nutrition survey
BMI: Body mass index
2SRI: Two-stage residual inclusion
2SLS: Two-stage least squares
